# Supplementary material for: E.PathDash, pathway activation analysis of publicly available pathogen gene expression data
Source: mSystems. 2024 Oct 18;9(11):e01030-24. doi: 10.1128/msystems.01030-24 (PMC11575265; doi:10.1128/msystems.01030-24)
Supplement: Supplemental material — Figures S1-S7 and Tables S1, S3, and S4. [file msystems.01030-24-s0001.pdf]

E.PathDash, pathway activation analysis of public cystic fibrosis pathogen gene expression data

Supplementary Information

Table S1. 1
Table S2. 2
Table S3. 2
Table S4. 2
Figure S1 3
Figure S2 4
Figure S3 5
Figure S4 6
Figure S5 7
Figure S6 8
Figure S7 9

1

Name	File Type	Contents	Access Point
Raw gene count data	zip	Matrix of gene counts for each dataset, design matrix for study samples	Study Explorer
Differential gene expression data	csv	Results of differential gene expression analysis for selected dataset and treatment comparison (gene, logFC, p-value)	Study Explorer
Significant KEGG pathways box plot	png	Box plot of distributions of gene logFC values for significantly expressed KEGG pathways for selected datasets and treatment comparison	Study Explorer
Significant GO terms box plot	png	Box plot of distributions of gene logFC values for significantly expressed GO terms for selected dataset and treatment comparison	Study Explorer
KEGG pathway analysis table	csv	Table of all KEGG pathways analyzed in pathway analysis (binomial test statistic, median gene logFC, p-value, FDR corrected p-value)	Study Explorer
GO term analysis table	csv	Table of all GO terms analyzed in pathway analysis (binomial test statistic, median gene logFC, p-value, FDR corrected p-value)	Study Explorer
KEGG pathway datasets table	csv	Table of all datasets and treatment comparisons with significant expression of selected KEGG pathway (study identifier, treatment comparison, median gene logFC)	KEGG Pathway Explorer
KEGG pathway volcano plot	png	Plot of logFC value and log-transformed p-value for genes along selected KEGG pathway in selected dataset and treatment comparison	KEGG Pathway Explorer
Differential expression of KEGG pathway genes	csv	LogFC and p-values for genes along selected KEGG pathway	KEGG Pathway Explorer
GO term datasets table	csv	Table of all datasets and treatment comparisons with significant expression of selected GO term (study identifier, treatment comparison, median gene logFC)	GO Term Explorer
GO term volcano plot	png	Plot of logFC value and log-transformed p-value for genes along selected GO term in selected dataset and treatment comparison	GO Term Explorer
Differential expression of GO term genes	csv	LogFC and p-values for genes along selected GO term	GO Term Explorer
Study comparison bar chart	png	Bar plot of median gene logFC values for each sample comparison within selected study for selected KEGG pathway or GO term	Study Comparison

Table S1.

Downloadable content available in E.PathDash. “Access Point” refers to the page of the application where the content can be accessed.

Table S2.

LogFC and p-values for genes in the propanoate metabolism KEGG pathway for *P. aeruginosa* grown in co-culture with WT *C. albicans* compared to monoculture and for *P. aeruginosa* grown with *adh1*Δ/Δ *C. albicans* compared to WT *C. albicans*. Gene and protein names as well as other annotation information retrieved from uniport.org based on uniport IDs.

Table S2 is available in a separate supplementary file titled "Supplemental_Table_2.xlsx"

Pathogen	Citation (Reference #)	Summary
Pseudomonas aeruginosa	Murphy T.F. et al 2008 (6), Fischer A. J. et al 2021 (7), Li K. et al 2020 (8), Willner D.L. et al. 2013 (10), Malhorta S. et al. 2019 (28), Eklof J. et al. 2020 (32)	*Pseudomonas aeruginosa* (P.a.) in COPD, co-infection with *Staphylococcus aureus* and *Streptococcus* in CF airway, association with bronchiolities obliterans syndrome in the CF lung, P.a. virulence in the CF lung
Staphylococcus aureus	Fischer A. J. et al 2021 (7), Hilty M. et al. 2010 (14) , Gangell C. et al. 2011 (26), Pillarisetti N. et al. 2011 (27), Rubinstein E. et al. 2008 (31), Purves J. et al. 2022 (70)	Co-infection with *Pseudomonas aeruginosa* in CF airway, role in pediatric asthma, association with increased lung inflammation in children with CF, role in hospital-acquired pneumonia, air pollution driven *S. aeurus* infection
Bacteroides thetaiotaomicron	Dickson R.P. et al. 2016 (12), Guo Y. et al. 2023 (13)	*Bacteroides* in acute respiratory disease syndrome and lung adenocarcinoma
Streptococcus sanguinis	Li K. et al. 2020 (8), Filkins L.M. et al. 2012 (9), Erb-Downward J.R. et al 2011 (11), Guo Y. et al. 2023 (13), Borewicz K. et al. 2013 (15)	*Streptoccocus* in competition with *Pseudomonas* in CF airway, promoting stability in the CF lung, role in lung adenocarcinoma, transplant lung microbiome

Table S3.

Summary of literature supporting relevance of *Pseudomonas aeruginosa, Staphylococcus aureus, Streptococcus sanguinis, and Bacteroides thetaiotaomicron* in respiratory disease research. All references are cited in the full manuscript and above include their corresponding reference numbers in the References section.

GSE Accession	Pathway	Comparison	Median gene logFC	Adjusted p-value
GSE124385	Sulfur metabolism	WT HOCl vs WT untreated	2.66	0.01
GSE124385	Sulfur metabolism	WT HOSCN vs WT untreated	1.26	0.01
GSE142448	Fatty acid metabolism	Manuka honey vs untreated	-0.34	0.006
GSE142448	Quorum sensing	Manuka honey vs untreated	-0.9	<0.001

Table S4.

Sample of datasets, identified by GSE accession, in which findings from the corresponding publications were identified by the E.PathDash pathway analysis pipeline. For each pathway and comparison listed in the table, the study identified the same biological processes activated/repressed in the same treatment comparison.

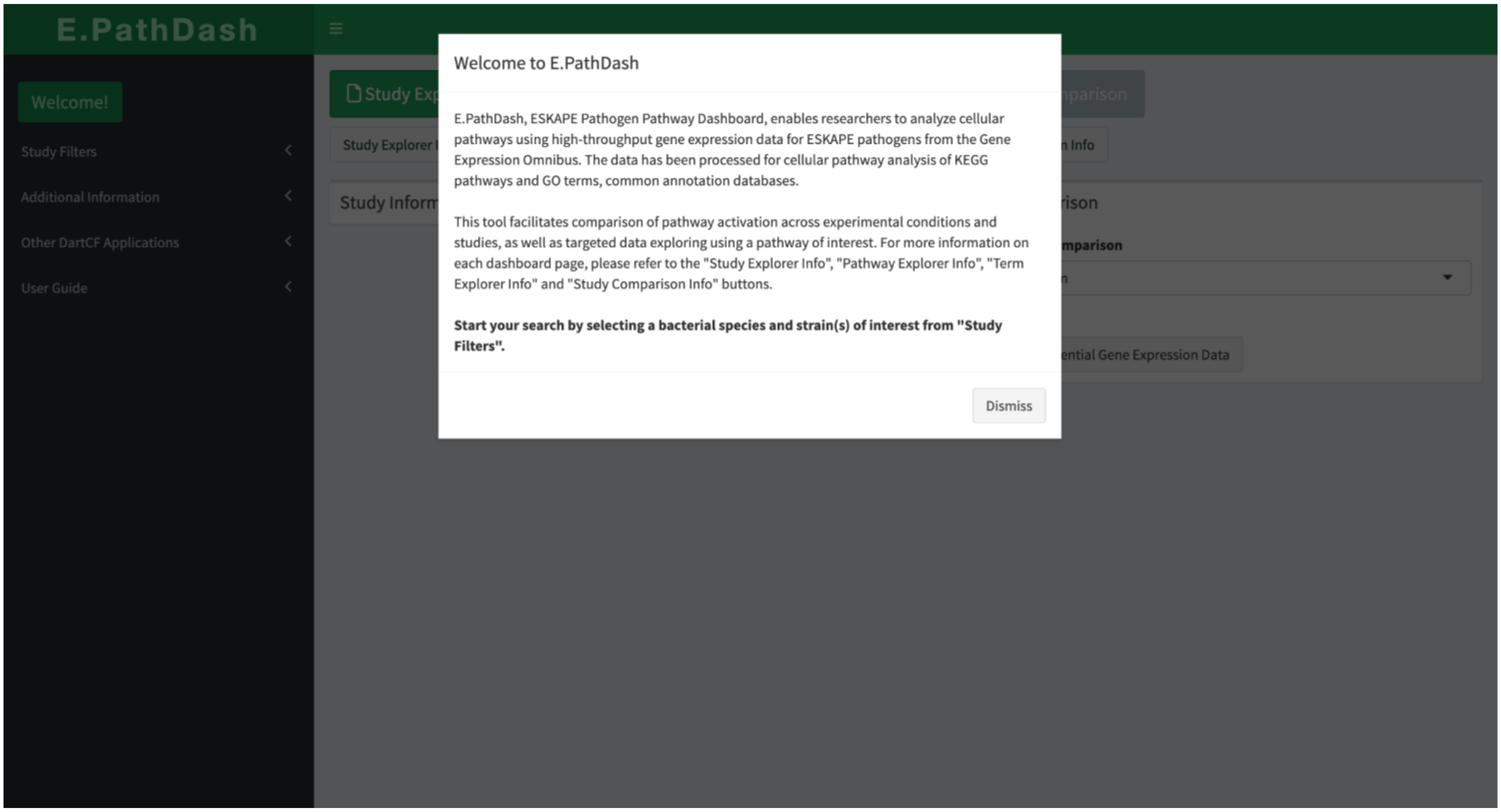

Figure S1.

Zoomed-in image of figure 2, screen 1 (landing page)

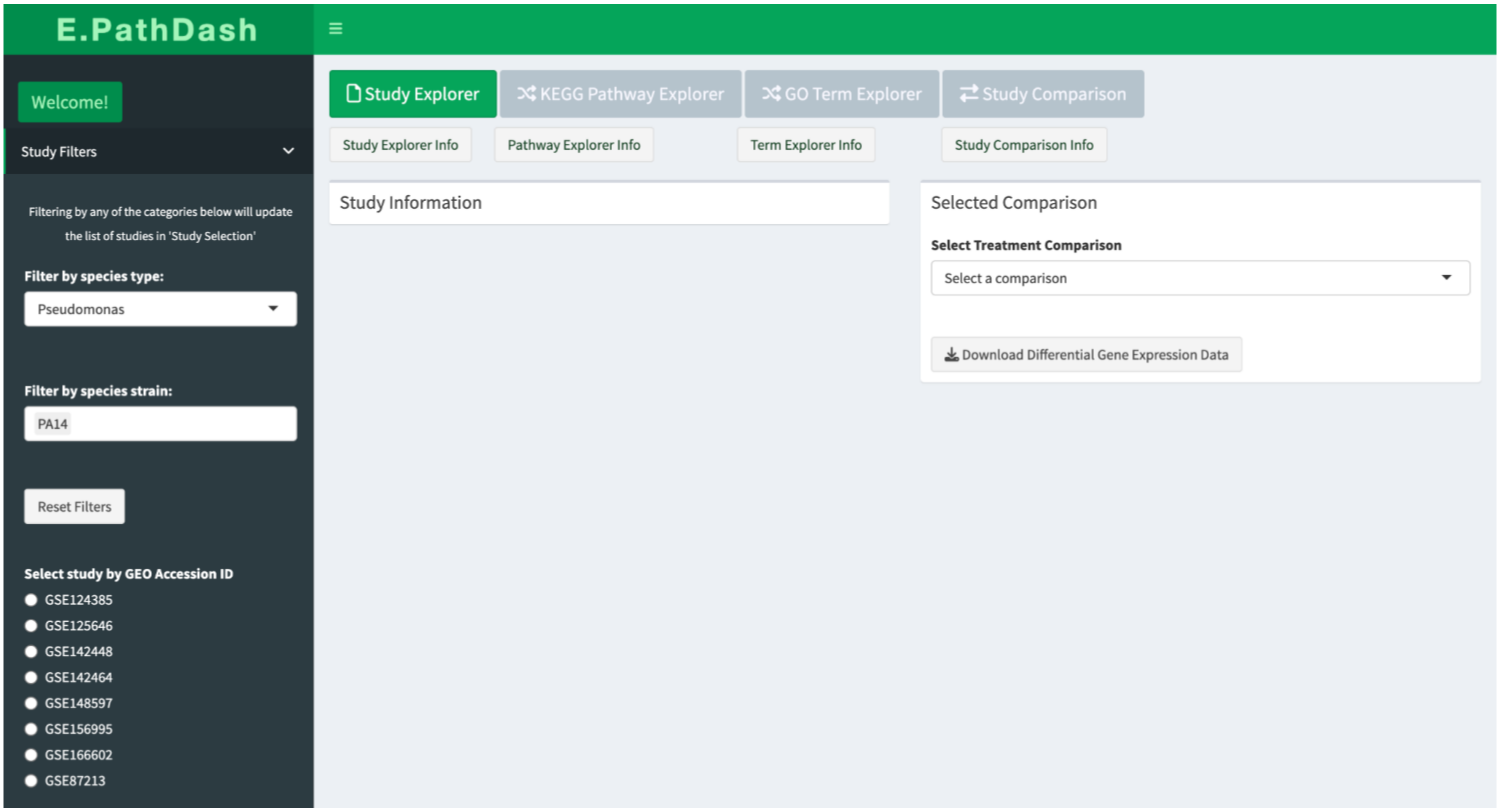

Figure S2.

Zoomed-in image of figure 2, screen 2 (filtering side panel)

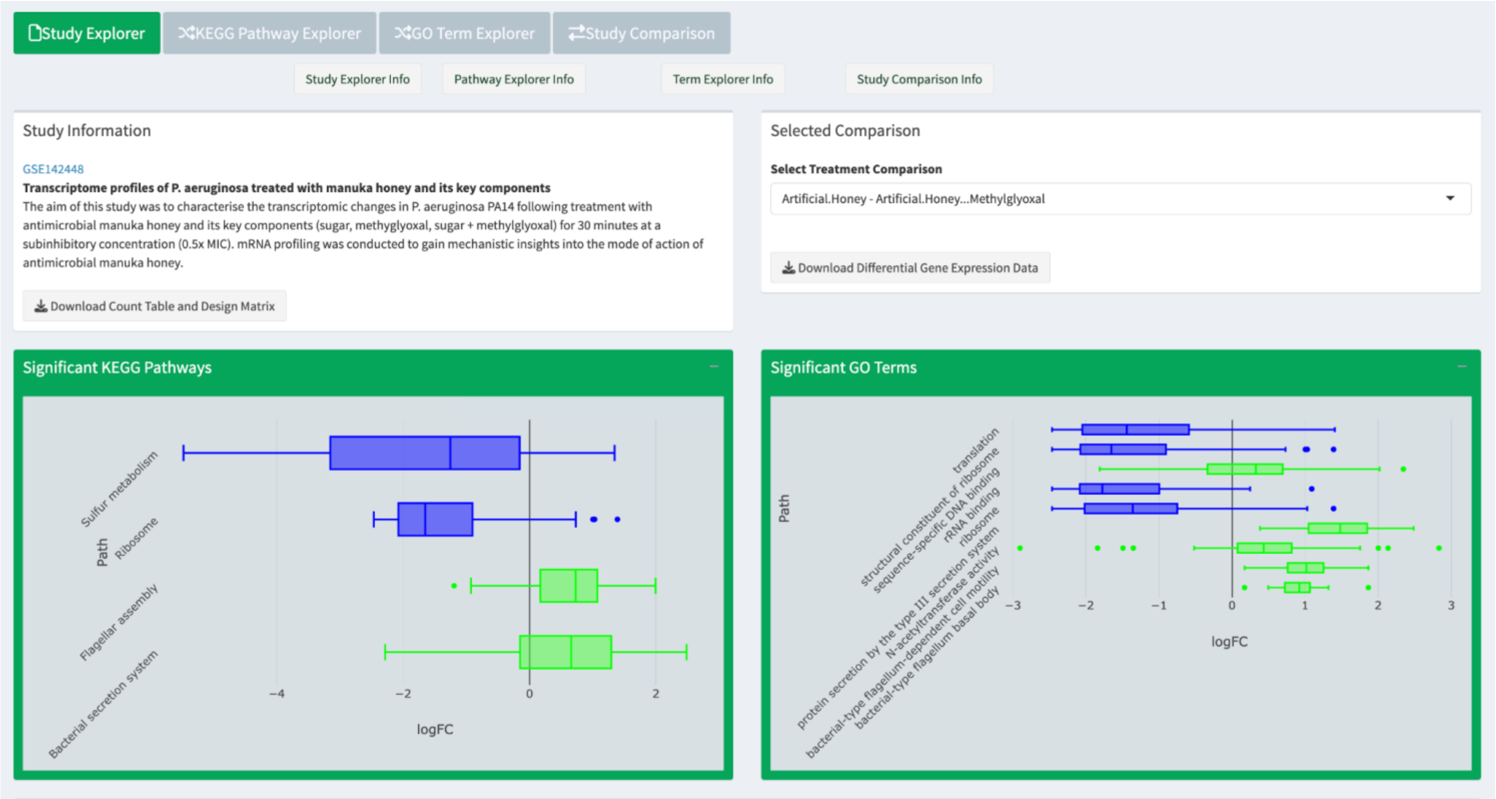

Figure S3.

Zoomed-in image of figure 2, screen 3 (Study Explorer page)

KEGG Pathways

Show 10 entries Search:

Path	Binomial Test Est.	Median gene logFC	P-value	FDR
2-Oxocarboxylic acid metabolism	0.45	-0.05	0.720100131817163	0.9784375
Alanine, aspartate and glutamate metabolism	0.24	-0.43	0.00256320799235255	0.0517768014455215
Amino sugar and nucleotide sugar metabolism	0.56	0.26	0.60759136127308	0.9784375
Aminoacyl-tRNA biosynthesis	0.3	-0.4	0.0522389858961105	0.378596987043109
Aminobenzoate degradation	0.44	-0.19	0.803619384765625	1
Arginine and proline metabolism	0.39	-0.46	0.160779601811988	0.667080983785647
Arginine biosynthesis	0.48	-0.04	1	1
Ascorbate and aldarate metabolism	0.29	-0.56	0.453125	0.944110240255084
Bacterial chemotaxis	0.65	0.37	0.0594633752537703	0.40038672670872
Bacterial secretion system	0.67	0.66	0.000924644411771929	0.0233472713972412

Showing 1 to 10 of 101 entries

Previous 1 2 3 4 5 … 11 Next

Download Table

GO Terms

Show 10 entries Search:

GO Term	Binomial Test Est.	Median gene logFC	P-value	FDR
'de novo' IMP biosynthetic process	0.43	-0.21	0.79052734375	1
'de novo' UMP biosynthetic process	0.57	0.05	1	1
2 iron, 2 sulfur cluster binding	0.42	-0.04	0.473129659891129	0.93698226762753
3-deoxy-7-phosphoheptulonate synthase activity	0.4	-0.18	1	1
3-oxoacyl-[acyl-carrier-protein] synthase activity	0.71	0.3	0.453125	0.918086547851563
3'-5' exonuclease activity	0.25	-0.34	0.2890625	0.840363968318158

Figure S4.

Zoomed-in image of pathway analysis tables on Study Explorer page. Not shown in figure 2.

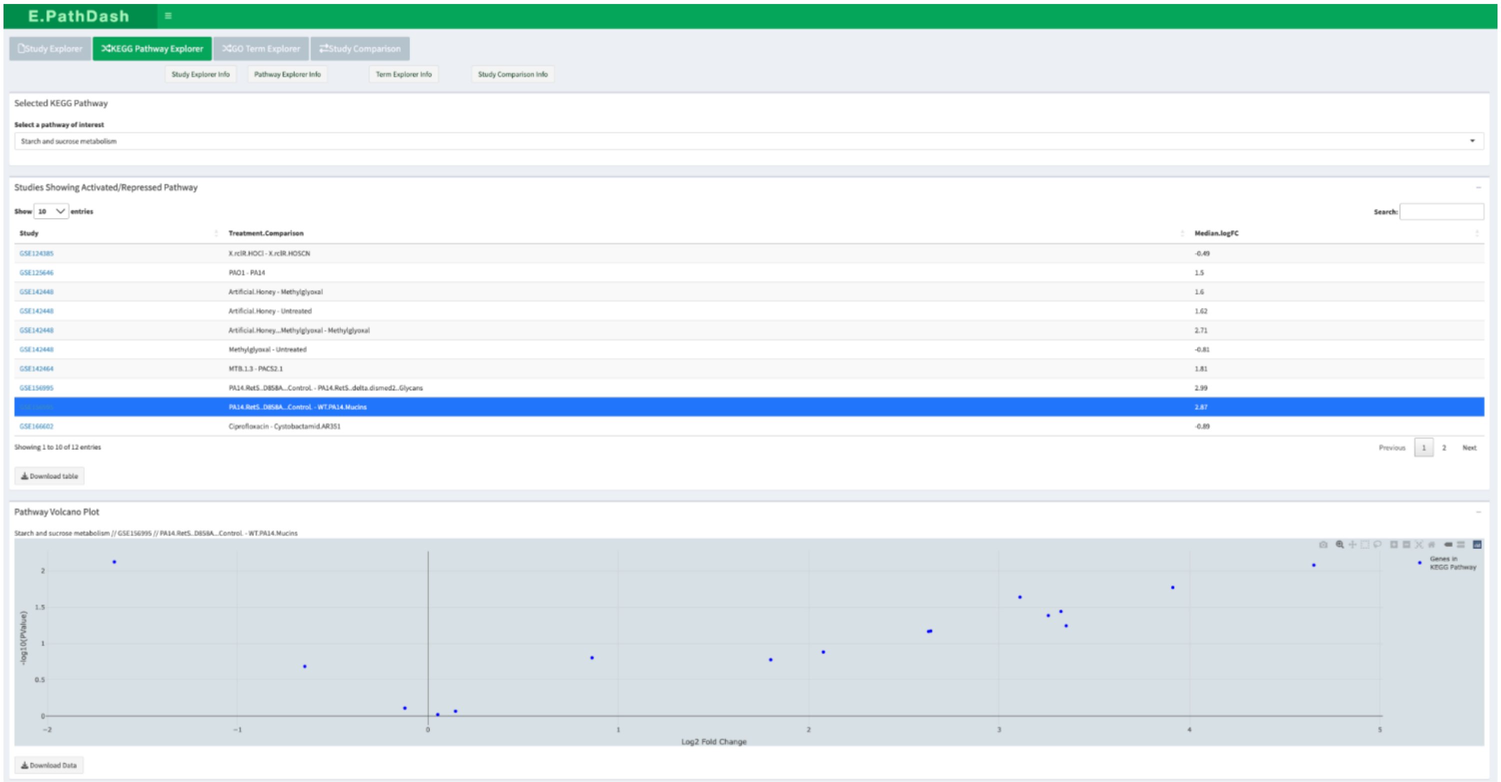

Figure S5.

Zoomed-in image of figure 2, screen 4 (KEGG Pathway Explorer page)

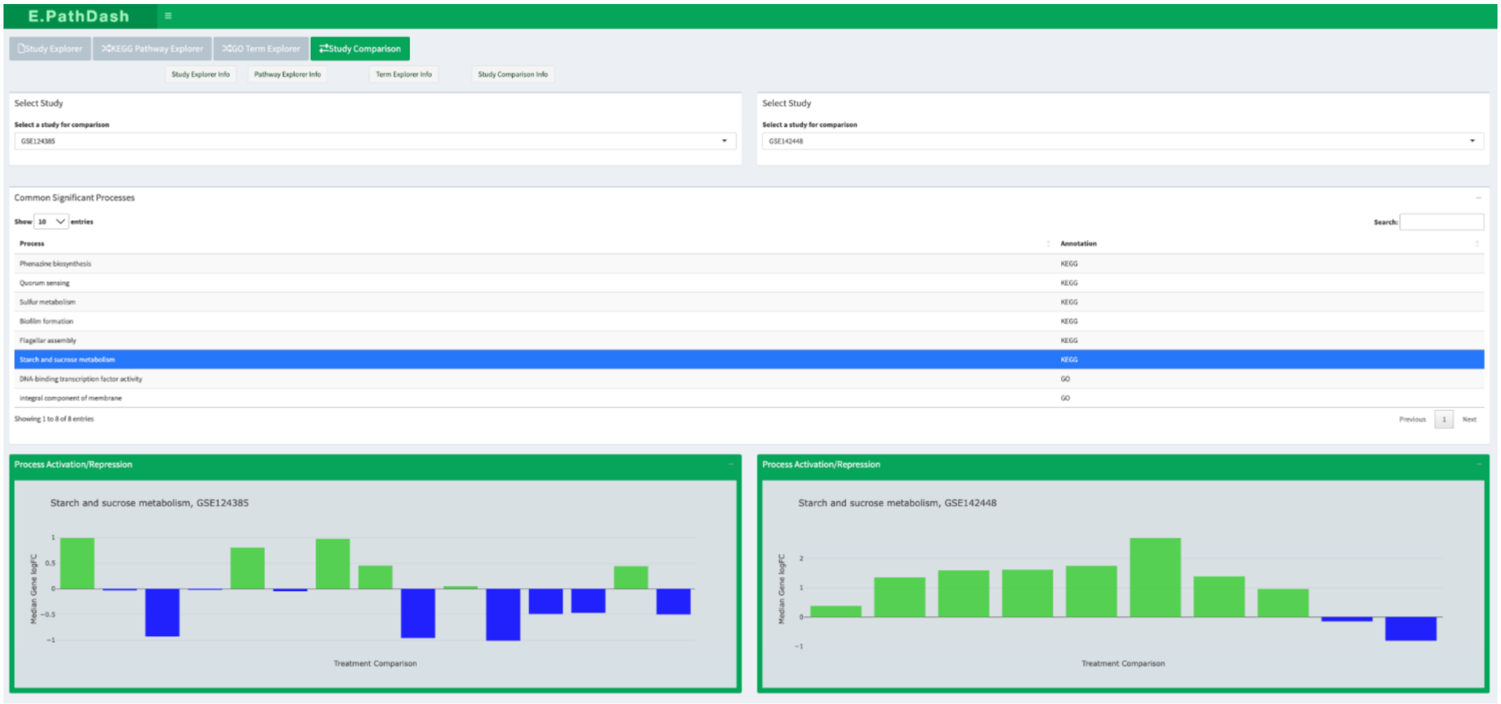

Figure S6.

Zoomed-in image of figure 2, screen 5 (Study Comparison page)

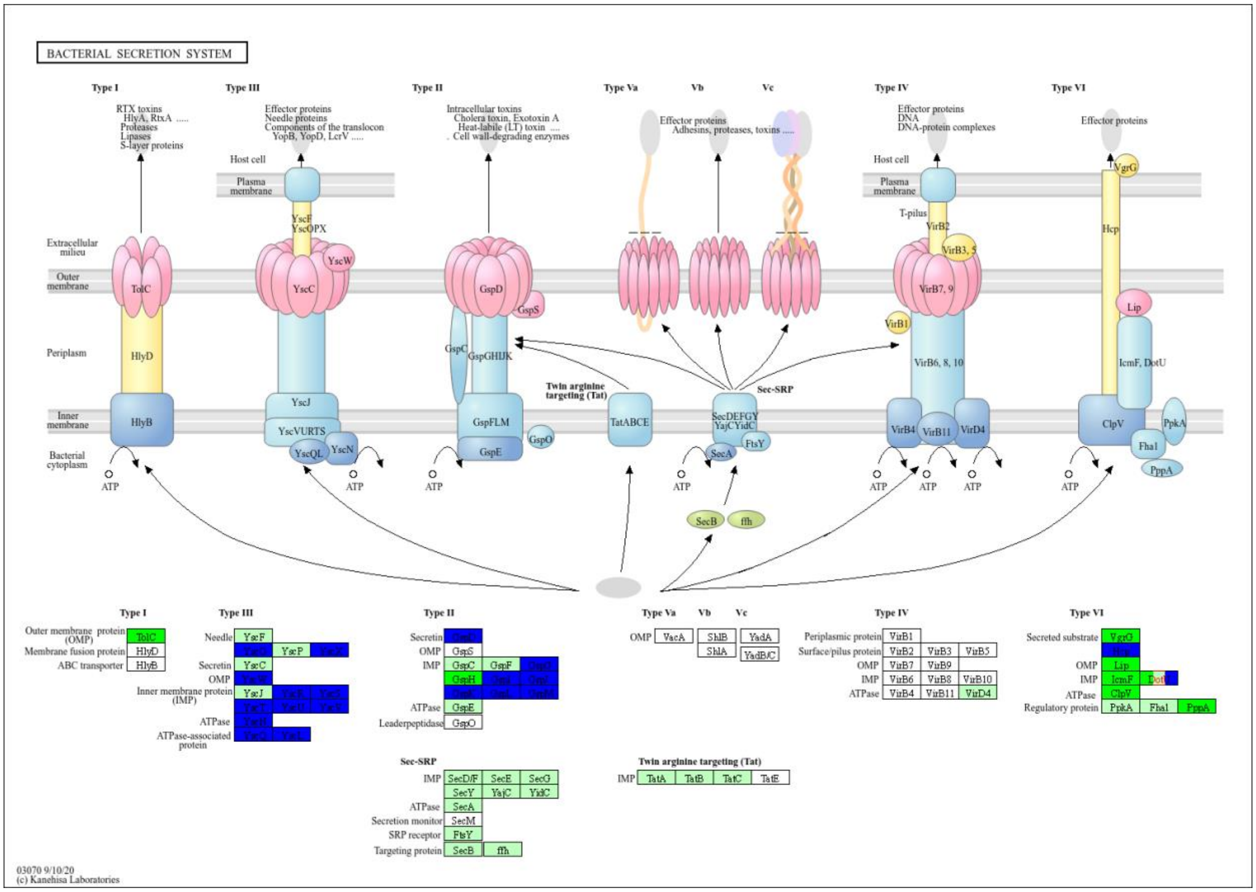

Figure S7.
KEGG pathway map showing differentially expressed genes between the untreated and hypochlorous acid (HOCl) conditions of WT *Pseudomonas aeruginosa* cells in study GSE124285 (Farrant et al.). Blue indicates the genes are repressed in the untreated cells compared to the HOCl condition, or in other words enriched in the HOCl condition. Green indicates the genes are enriched in the untreated condition compared to the HOCl condition. The genes are colored by the E.PathDash application according to their logFC values. The published study associated with this dataset, by Farrant et al., reported that exposure to HOCl enriched genes involved in the type III secretion system, which we can see represented here.
